# Practical MR mammography – high-resolution MRI of the breast

**DOI:** 10.1002/jmrs.12

**Published:** 2013-05-28

**Authors:** Dominic Kennedy

**Affiliations:** Queensland X-Ray, Greenslopes Private HospitalNewdegate Street, Greenslopes, QLD 4120, Australia

*Practical MR Mammography* is the second edition of this book – the first was published 8 years previously. There have been considerable changes in breast magnetic resonance imaging (MRI) over this time, and these changes are reflected in this new edition, with completely rewritten chapters including technique, methodology, lesion analysis, biopsy, quality assurance, and high-quality images. Uwe Fischer is obviously passionate about MR mammography which is reflected in this practical and comprehensive book.

The contents of the book are logically and sequentially ordered from patient preparation to imaging to diagnosing the lesions. His summary of the technique and methods used to image the breast is comprehensive. Each section, such as imaging during a menstrual cycle, patient positioning, sequences, and contrast agents, all contain clear and concise explanations.

The spatial resolution one should expect with MR mammography (2013 – 0.6 to 1.2 mm slice thickness) is greater than that stated in this book, and an explanation of spatial resolution in terms of pixel size would be better as it takes into account the slice thickness, field of view, and image matrix.

In terms of MR imaging approaches, it would be helpful to have other imaging approaches detailed, that is, the dynamic “interview” approach using an ultra–high-resolution sagittal scan interspersed between axial dynamic scans. A limited explanation of imaging the breast with 3T is evident and would be a useful addition in any future editions.

Extremely useful are the “exclamation points” in each section throughout the book providing the reader with “take-home” (important) messages.

Assessment of each of the MR pulse sequences (T1, T2) is provided and diagnostic criteria is used to help the reader interpret the images and structures within the breast in a logical and clear manner.

Explanations throughout the text are accompanied by high-quality images to explain and illustrate the points being made. An excellent explanation of the contrast enhancement patterns of lesions, based on the ACR BI-RADS-MRI Lexicon, is approached and explained systematically. Artefacts seen in breast MR are explained and reasons for their appearance are given, which will help the reader understand problems that can occur when imaging the breast and how to correct for them.

Easily accessible sections describing normal findings in breast MR, benign changes, postoperative/posttraumatic changes, borderline lesions, intraductal carcinomas, malignant changes, lymph nodes, and breast prosthesis are examined in the same clear, methodological manner. A brief explanation of the lesion, MR characteristics, image examples together with dynamic curve analysis are provided for each lesion. Reference tables are provided which explain the clinical, mammographic, and sonographic appearance of these lesions to go with the MR explanations.

One of the most common uses of breast MR is as a problem solver in ambiguous findings. Other indications such as differentiation between scar and local recurrence after breast conserving therapy; the search for cancers of unknown origin; preoperative local staging; and monitoring for neoadjuvant chemotherapy are detailed, accompanied by the explanation as to why MRI is the preferred imaging solution in these situations.

MR-guided biopsy and localization is detailed with a step-by-step checklist to follow when performing these procedures. A brief explanation of the different biopsy coils and the different targeting techniques is provided. However, a more comprehensive explanation of the different targeting techniques, biopsy coils, vacuum biopsy equipment, and the advantages and disadvantages of each would be useful in assisting imaging staff starting interventions to be more confident in choosing the right equipment.

The section on differential diagnosis and strategy gives pertinent advice on commonly encountered situations, such as solitary focus, multiple foci, non–mass-like lesions, and outlines strategies for management of these lesions. Of particular relevance is the explanation of when to recommend biopsy and follow-up interval strategies for a variety of lesion types. These strategies are detailed in clear, easy-to-follow tables. Examples of written reports for breast MR are provided to help breast imagers beginning in this dynamic field.

This book would be an excellent addition as a resource for both imaging technologists and radiologists. As a practical atlas for radiologists to refer to for image examples of certain pathologies and guidance for differential diagnosis, it would be a beneficial investment.

